# Investigating the effects of substrate morphology and experimental conditions on the enzymatic hydrolysis of lignocellulosic biomass through modeling

**DOI:** 10.1186/s13068-021-01920-2

**Published:** 2021-04-26

**Authors:** Jessica C. Rohrbach, Jeremy S. Luterbacher

**Affiliations:** Laboratory of Sustainable and Catalytic Processing, Institute of Chemical Sciences and Engineering, École Polytechnique Fédérale de Lausanne (EPFL), CH-1015 Lausanne, Switzerland

**Keywords:** Modeling, Lignocellulosic biomass, Cellulase, Hydrolysis, Porosity, Mass transfer

## Abstract

**Background:**

Understanding how the digestibility of lignocellulosic biomass is affected by its morphology is essential to design efficient processes for biomass deconstruction. In this study, we used a model based on a set of partial differential equations describing the evolution of the substrate morphology to investigate the interplay between experimental conditions and the physical characteristics of biomass particles as the reaction proceeds. Our model carefully considers the overall quantity of cellulase present in the hydrolysis mixture and explores its interplay with the available accessible cellulose surface.

**Results:**

Exploring the effect of various experimental and structural parameters highlighted the significant role of internal mass transfer as the substrate size increases and/or the enzyme loading decreases. In such cases, diffusion of cellulases to the available cellulose surface limits the rate of glucose release. We notably see that increasing biomass loading, while keeping enzyme loading constant should be favored for both small- (*R* < 300 $\mu m$) and middle-ranged (300 < *R* < 1000 $\mu m$) substrates to enhance enzyme diffusion while minimizing the use of enzymes. In such cases, working at enzyme loadings exceeding the full coverage of the cellulose surface (i.e. *e*_*I*_>1) does not bring a significant benefit. For larger particles (*R* > 1000 $\mu m$), increases in biomass loading do not offset the significant internal mass transfer limitations, but high enzyme loadings improve enzyme penetration by maintaining a high concentration gradient within the particle. We also confirm the well-known importance of cellulose accessibility, which increases with pretreatment.

**Conclusions:**

Based on the developed model, we are able to propose several design criteria for deconstruction process. Importantly, we highlight the crucial role of adjusting the enzyme and biomass loading to the wood particle size and accessible cellulose surface to maintain a strong concentration gradient, while avoiding unnecessary excess in cellulase loading. Theory-based approaches that explicitly consider the entire lignocellulose particle structure can be used to clearly identify the relative importance of bottlenecks during the biomass deconstruction process, and serve as a framework to build on more detailed cellulase mechanisms.

**Supplementary Information:**

The online version contains supplementary material available at 10.1186/s13068-021-01920-2.

## Background

Faced with global warming, one of society's main challenges for the twenty-first century is to develop sustainable alternatives to current non-renewable and carbon-emitting resources. While lignocellulosic biomass appears to be an attractive alternative carbon source to fossil feedstocks as low-cost biomass residues are widely available [1], biomass conversion to fuels and chemicals still requires further development to become economically feasible [2]. An important bottleneck for the biochemical transformation of lignocellulosic substrates into value-added chemicals is the initial hydrolysis of complex carbohydrates to simple sugars [3], which can further be processed to obtain the desired final products [4, 5]. The high-cost of this procedure is related to the resulting yields of monosaccharides, the rate of hydrolysis, and quantity of the enzymes required. These factors are always heavily influenced by the physicochemical, structural and compositional properties of the substrate itself, as well as its interactions with enzymes.

In their untreated native state, biomass polysaccharides have a low digestibility. For this reason, a pretreatment step is required to disrupt the lignin and hemicellulose matrix hindering cellulases’ access to the cellulose fibers and improve depolymerization rates [6–9]. Particle size reduction, porosity changes, disruption of lignin structure as well as cellulose accessibility, crystallinity, degree of polymerization, shielding by hemicellulose and packing of the cellulose fibers have all been highlighted as parameters affecting digestibility to various degree and at different stages of the hydrolytic process [10–12]. Along with the impact of biomass structure, many other mechanisms have been reported to explain limitations in the efficiency of the digestive process including: enzymes’ intrinsic thermal sensitivity [13, 14], inhibition from both hydrolysis products or residual compounds from the pretreatment step [15–17], slowing kinetics through pore entrapment and/or surface jamming [18, 19], reduced processivity [20–22] as well as unproductive binding to lignin [23, 24], and inadequacy of the enzyme cocktail composition to promote synergism [25, 26]. Two notable phenomena that are well known but poorly understood, at least quantitatively, are the decrease in hydrolysis rates observed as the reaction proceeds [27–30] and the similar decrease in rates with increased solid loading [31, 32]. Given their importance for industrial scale-up, this lack of understanding impedes the implementation of economically viable biomass-to-biofuels pathways. Of the factors influencing enzymatic deconstruction, no single one can fully explain hydrolysis trends. Studying enzymatic hydrolysis is notably complicated (i) by the difficulty of experimentally investigating each factor independently, as targeted modifications of one parameter usually impact the others, and (ii) by the experimental observations being highly dependent on the substrate native structure and treatment history, as well as the composition of the enzyme cocktail that is used.

In this context, a theory-based model coupled to experimental observations could help untangle the complex relationship between substrate specificities and digestibility and help develop design principles for both pretreatment and enzyme cocktail design. Significant modeling efforts have focused on detailed cellulase–cellulose interactions describing the hydrolysis kinetics of cellulosic substrates and have highlighted the role of cellulose ultrastructure and its evolution over the course of hydrolysis as well as on enzyme synergism [33–37]. Other modeling strategies have incorporated the effect of the entire lignocellulosic structure on the biomass deconstruction, mainly in an implicit way. Specifically, the impact of mass transfer arising from biomass particle size and/or loading have been incorporated or considered through the use of empirically trained artificial neural networks [32], addition of corrective kinetic terms in fractal-like kinetic modeling [38], or phenomenological description of the hydrolytic process [39].

**Fig. 1:**
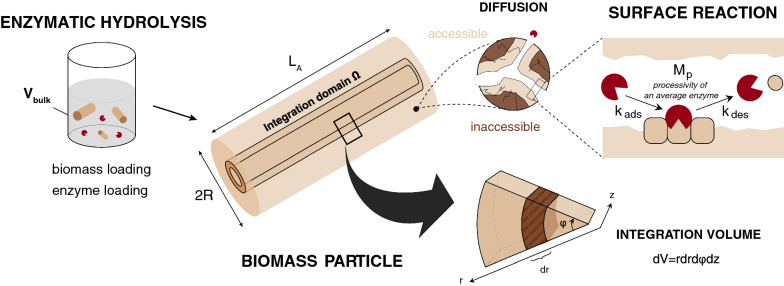
Schematic representation of the assumed biomass geometry and enzymatic hydrolysis mechanism. Only a fraction of the biomass particle, containing all pores accessible to cellulases, is subjected to hydrolysis. Subsequently to their diffusion to the binding sites located at the pore wall, enzymes bound to cellulose fibers and catalyze their depolymerization (surface reaction). The integration volume is given by ${\varvec{d}}{\varvec{V}}={\varvec{r}}{\varvec{d}}{\varvec{r}}{\varvec{d}}\boldsymbol{\varphi }{\varvec{d}}{\varvec{z}}$

Here, we use a diffusion–reaction model describing the enzymatic hydrolysis of lignocellulosic biomass particles with a focus on the substrate’s physical evolution to evaluate the interplay between cellulose accessibility to cellulase and glucose release (Fig. 1). Notably, we evaluate the effects of experimental conditions, including enzyme- and biomass-loadings, on the reaction rate. While the morphology of the biomass particle (i.e. overall physical properties of the substrate comprising porosity, component distribution and size) is explicitly incorporated in the model, the enzymatic action is reduced to a simple time-dependent Langmuir isotherm in which the removal soluble sugars is accounted for during the desorption step. Cellulose hydrolysis is expressed through a lumped parameter that averages several specific cellulase mechanisms, such as processivity, adsorption mechanisms on the cellulose surface and the effect of different enzymes including synergistic effects. While such diffusion–reaction systems in evolving porous media have been subject of extensive research efforts in several domains—including the modeling of catalyst deactivation [40] or the study of mineral deposition in hydrology/geology [41] to only cite a few—we used a similar formalism here to describe the enzymatic hydrolysis of lignocellulosic biomass. Our goal was to provide a detailed modeling framework for the structural effects controlling enzymatic hydrolysis of lignocellulosic biomass. This approach is meant to complement more detailed kinetic models for pure cellulosic substrates, notably by highlighting possible hindrances stemming from the substrate’s morphology in addition to those arising from the cellulose itself.

## Results/discussion

### Modeling framework

The detailed derivation of the model used in this study can be found in the “Methods” section, which is based on a previously published model [42]. Description of all parameters and variables used in the model can be found in Table 1. In short, we model the enzymatic hydrolysis of a porous, non-shrinking cylindrical lignocellulosic particle in batch conditions through a system of coupled partial differential equations (see Additional file 1: A0). This assumption is supported by previous imaging studies that have observed hollowing out particles by in situ confocal microscopy during hydrolysis [42, 43]. These equations account for diffusional effects that are influenced by variable enzyme- and biomass-loadings. Experimental inputs include the physical characterization of the substrate (porosity, particle size, composition, digestibility). The physical description of the substrate is complemented by an additional fitted parameter representing the pore network tortuosity τ. These inputs and fitted parameter allow for the determination of the substrate’s cellulose accessibility to cellulases over the course of the hydrolysis, i.e. the number of available adsorption sites on the accessible cellulose surface. Cellulose hydrolysis is implicitly expressed through a fitted parameter ${M}_{p}$ representing the glucose release per enzyme binding cycle. Thus, all cellulose adsorption sites calculated based on the aforementioned experimental measurements are considered equal. Consequently, ${M}_{p}$ accounts indistinctly for effects related to hydrolysis including: the distinct type of enzymes in the cocktail, synergism, individual mechanisms of these cellulases and local specificities in the cellulose structure (enzyme processivity, differentiated adsorption sites, cellulose structural heterogeneities, enzyme synergism, etc.) and, as such, represents an overall measure of the cellulose’s susceptibility to be digested by a given enzyme cocktail. Mathematically, we can demonstrate that this parameter is equivalent to the average intrinsic processivity on cellulose for a single enzyme within the enzyme cocktail. Interestingly, the values of M_p_ that were estimated here, systematically lie between those reported for apparent- and intrinsic-processivity for single exoglucanases on crystalline cellulose surfaces (see Additional file 1: A1). This result is expected given that the average processivity of a cocktail on real biomass is expected to be higher than that measured of a single endoglucanase (apparent processivity), but lower than the value on an ideal polymer (intrinsic processivity).

**Table 1:** List of symbols. Dependent variables (Dep. Var.) refer to variables that are defined by or are calculated directly from experimental data

Symbol	Description	Value	Units
${b}_{l}$	Biomass loading	Dep. Var.	[g/cm^3^]
${C}_{E,0}$	Initial bulk enzyme concentration	Dep. Var.	[mol/cm^3^]
${C}_{E}^{F}(r,t)$	Enzyme concentration in pores or bulk (r = R)	Dep. Var.	[mol/cm^3^]
${C}_{E}^{S}(r,t)$	Enzyme concentration adsorbed at the cellulose surface per total cylinder volume V(r)	Dep. Var.	[mol/cm^3^]
${C}_{E,max}^{S}(r,t)$	Maximum enzyme concentration adsorbed at the cellulose surface per total cylinder volume V(r)	Dep. Var.	[mol/cm^3^]
${D}_{E}^{eff}(r,t)$	Effective cellulase diffusivity in pores	Dep. Var.	[cm^2^/min]
${\stackrel{-}{D}}_{E}^{pore}$	Average cellulase diffusivity in pores	Dep. Var	[cm^2^/min]
${D}_{E}$	Time independent part of the effective cellulase diffusivity in pores	Dep. Var.	[cm^2^/min]
${e}_{l}$	Enzyme loading in terms of enzyme:initial binding sites molar ratio	Dep. Var	[mol_E_/mol_Sites_]
*H*_*glu*_	Hydrolysis factor for glucose	0.9	[MM_cellulose_/MM_glucose_]
${k}_{ads}$	Cellulase surface adsorption rate [60]	$3 \cdot {10^{10}}$	[cm^3^/(mol $\cdot$ min)]
${k}_{des}$	Cellulase surface desorption rate [42, 43]	$0.068$	[1/min]
${M}_{p}$	Average moles of glucose liberated in solution per mole of cellulase during one binding-reaction cycle	Fitted var.	[mol_glucose_/mol_desorbing enzyme_]
${MM}_{glu}$	Molar mass of glucose	180	[g/mol]
$n$	Grid size	Dep. Var.	[−]
${n}_{E,0}$	Total number of moles of enzyme in the system	Dep. Var.	[mol]
$r$	Radial distance	Indep. Var.	[cm]
$R$	Particle radius	Dep. Var.	[cm]
${S}_{c}$	Pore cellulose surface	Dep. Var	[cm^2^]
${S}_{cyl}$	Outer particle surface	Dep. Var.	[cm^2^]
$t$	Time	Indep. Var.	[min]
${V}_{B}$	Volume of bulk solution	Dep. Var.	[cm^3^]
$\varepsilon (r,t)$	Porosity	Dep. Var.	[cm^3^_pore_/ cm^3^_biomass_]
${\varepsilon }_{0}$	Initial porosity	Dep. Var.	[cm^3^_pore_/ cm^3^_biomass_]
${\rho }_{C}^{IV}$	Density of cellulose including void	Dep. Var.	[g/cm^3^]
$\varphi$	Angular coordinate	Indep. Var.	[−]
$\sigma$	Maximum cellulase surface concentration on cellulose [42, 43]	$2.1\cdot{10}^{-12}$	[mol/cm^2^]
$\tau$	Tortuosity	Fitted var.,	[−]
*z*	Longitudinal coordinate	Indep. Var.	[-]

The following analyses are focused on the early stage of hydrolysis, in which the substrate structural changes can be assumed to be unchanged aside from their evolution in porosity, which was shown to not be sufficient to fully explain the rate slow down [42, 43]. In addition, any consequences of enzyme deactivation mechanisms can similarly be assumed as minor. This allows us to accurately explore these initial effects before evaluating, in subsequent work, how changes in particle size and shape and effects of enzyme deactivation play a role in the rate slowdown that is usually observed following initial hydrolysis. The following section are organized as follows: first, we evaluate the effect of accounting for a finite number of enzymes in the system on a dataset taken from literature (referred to as DS1) and previously discussed within the previous model formulation [29, 42, 43]. We show the validity of our model to predict initial rates based on initial cellulose accessibility for this particular dataset (DS1). We then evaluate in silico how changes in experimental conditions would affect early hydrolysis rates for one of the substrates included in this dataset (DS1). Finally, we discuss how these in silico predictions predict new experimental data by generating a new dataset (referred as DS2) that covers conditions were in silico results predicted changes in hydrolysis rates. For sake of clarity, we refer throughout the text to the dataset extracted from literature as DS1 and the newly generated dataset as DS2.

### The relationship between enzyme loading and cellulose accessibility

**Fig. 2:**
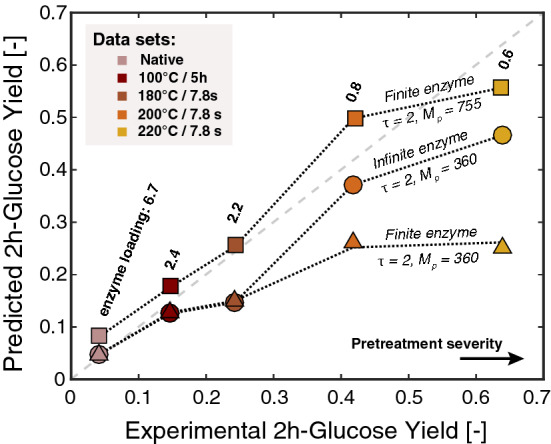
Comparison of predicted initial glucose yields to dataset DS1 taken from literature for native and acid-pretreated (1% acid) mixed hardwood at increasing degree of severity [29]. Predictions from both models—accounting for finite (triangle) or infinite (circle) enzyme loadings—are reported using fitted parameters unchanged from the infinite enzyme fit (${M}_{p}$ = 360, *τ* = 2). Also shown are the early glucose yield predictions considering finite enzyme with refitted parameter (${M}_{p}$ = 755, $\tau=2$) obtained by least-square fittings over the whole range of substrates (square). Enzyme loadings in terms of enzyme:initial binding site molar ratio are shown for each substrate and correspond to an experimental enzyme:substrate mass ratio of 92.5 mg cellulase/g

In previous work [42, 43], not considering enzyme depletion in the bulk provided rather good predictions of initial yields and quantitatively confirmed the importance of surface accessibility as a key parameter on the hydrolysis. However, with the more complicated model developed here, we demonstrate that enzyme depletion can strongly impact the rate of cellulose hydrolysis especially for specific biomass structures (Fig. 2). We compare predictions from our previous work with those generated using the current improved model on the same dataset DS1 [29, 42, 43] (see Additional file 1: Table S1 in A2).

Using the fitted parameters (${M}_{p}=360$ and $\tau =2$) from the model assuming infinite enzyme loadings (no change in the bulk concentration of enzymes), accounting for enzyme depletion within the new framework worsens early yield predictions for the more severely pretreated hardwood, while generating similar results for mildly pretreated and native substrates (Fig. 2). For the latter, the initial excess cellulases in terms of adsorption sites (${e}_{l}>2$) makes the constant enzyme bulk concentration hypothesis reasonable. For more digestible substrates, the internal mass transfer is slowed by enzyme depletion in the bulk and to incomplete initial surface coverage, which significantly slows the initial glucose release compared to a case where no enzyme depletion is accounted for. As we will discuss below (see subsection “High enzyme- and biomass-loadings as drivers for enzyme penetration”), this fractional coverage of available cellulase binding sites can play a significant role in controlling the rate. Due to the difference in pretreatment severity and thus porosity, a significant difference exists across various substrates in number of adsorption sites. As a result, the molar ratio of enzyme:initial binding sites can vary significantly for the different substrates even if the ratio of enzyme:substrate mass is kept constant (0.925 mg/ml) [29] (see Additional file 1: Table S1 in A2) (Fig. 2). These differences, in turn, lead to significant variations in the maximum possible coverage of the enzymes at the start of the reaction, and thus can reduce the initial rate for cases where enzyme depletion leads to less than full coverage at the beginning of the reaction.

Improved predictions were obtained by refitting the parameters to the new model. The combined least-square fitting of both parameters on the whole set of data led to new values of ${M}_{p}=755$ glucan monomers liberated per binding cycle of one enzyme and $\tau$ = 2 (see Additional file 1: Figure S3 in A3). These parameters allowed for an accurate prediction of early glucose yields in good agreement with experimental observations (Fig. 2) (see Additional file 1: Figure S3 in A3). The significant increase of ${M}_{p}$ can be attributed to the previous overestimation of the number of enzyme present in the system. Assuming an infinite number of enzymes in silico when in reality a limited quantity was present lead to an underestimation of the hydrolytic capacity of the enzyme. Interestingly, the pore network complexity had an important impact on the predicted early glucose yields for the mildly pretreated substrates, while having a more minor influence on both native and more severely pretreated substrates (see Additional file 1: Figure S3/S4). In the case of the low accessibility extreme, the reaction rate was governed by a poor cellulose accessibility due to the low porosity for the native substrate. For the more severely pretreated wood samples, which was the highly accessible extreme, the rate was almost purely governed by the surface reaction rate and the pore network complexity played a fairly limited role.

Even though pore connectivity has been shown to increase (i.e. decreasing tortuosity) with the severity of acid-pretreatment of *populus* substrates (0.1 M sulfuric acid (SA) / 160 °C / 5–60 min) [44] and similar treatments increase cellulose digestibility [10–12], no clear trends were predicted by the fitting of $\tau$ and ${M}_{p}$ for individual substrates (see Figure S4 in Additional file 1:A3).

In addition, a single set of fitted parameters allowed us to accurately predict initial rates for a range of acid-diluted pretreated substrates, suggesting that similar modifications in the pore network structure and cellulose susceptibility to digestion occur independently to pretreatment severity (Fig. 2) (see Additional file 1: Figures S3/S4 in A3). However, this explanation should be treated cautiously, as no information on the particle size distribution after pretreatment was available and predictions were based on the size of the native substrate (with diameter of $25 \mu m$). Even though this important structural factor can play a significant role on early glucose yield (see section “Role of particle size reduction on internal mass transfer*”*), it seems unlikely to drastically shift predictions in this case, as the already small size of the native particle is expected to exhibit low diffusional resistance.

### High enzyme- and biomass-loadings as drivers for enzyme penetration

**Fig. 3:**
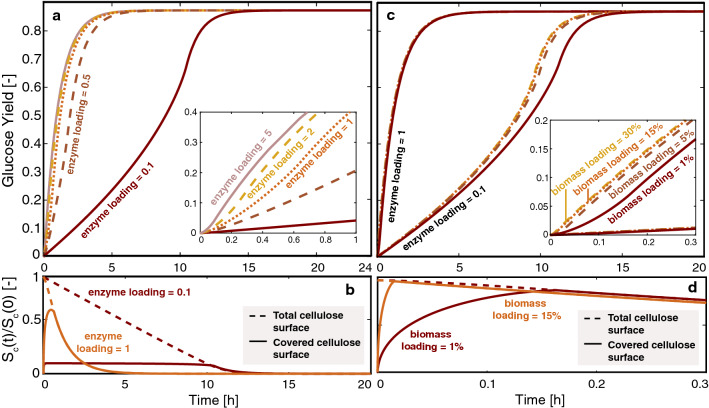
Predicted glucose yields and surface coverage with time for a substrate defined in dataset DS1, while varying experimental conditions in silico. Glucose yield as a function of time for **a** different enzyme loadings at a constant solid loading of 2%, and **c** different biomass loadings. **b**, **d** Illustrations of the progressive coverage of the cellulose surface for the chosen simulations: for **d**, the case where a molar ratio of enzyme:initial binding site of 1 was used

To evaluate the model’s predicted effect of experimental parameters on the course of the reaction, cellulose depolymerization was run in silico for relevant ranges of enzyme- and biomass-loading, using as model substrate an acid-pretreated mixed hardwood from dataset DS1 (dilute acid pretreatment (DAP) at 220 °C for 7.8 s) (Fig. 3). This substrate had a small particle size (assumed to be $25 \mu m$, due to sieving) with a high digestibility (> 80% glucose yield after 24 h).

For such small particles, both internal and external mass transfers are expected to be minimally limiting the rate (see Additional file 1: A4). Nevertheless, varying enzyme loadings for a fixed solid loading—in this case 2%—strongly affects the course of hydrolysis (Fig. 2). When the amount of cellulase is low compared to the number of adsorption sites (${e}_{l}<1$), a specific pattern in the glucose release emerges; up until the surface is completely covered with enzymes, the rate of cellulose degradation is limited by the number of adsorbed cellulases, which is controlled by the quantity of free enzymes in the pore lumen (Fig. 3a, b). Once the number of enzymes matches the available amount of adsorption sites in the system, the rate rapidly increases to match the maximum surface reaction rate. This rate only decreases once the quantity of remaining accessible cellulose decreases significantly, towards the end of hydrolysis. This initial rate transition becomes less noticeable with increasing enzyme loading, as the surface becomes saturated more rapidly.

For systems where the initial number of enzymes matches or exceeds the number of adsorption sites on the cellulose surface (${e}_{l}\ge 1$), further increasing the cellulase loading only slightly improves the rate of glucose release. This slight improvement in rate is due to the increase in enzyme concentration gradient in the particle within the 30 min of this multi-hour reaction. Glucose generation is almost solely reaction-limited for this particle size, as the entire available cellulose surface is rapidly covered leading to this small effect. Without deactivation mechanisms, the model shows that, at a certain point, working with large enzyme excesses does not improve performance. Importantly, the model allows to clearly extract diffusion effects from these other enzymatic phenomena, which we are currently implementing into the model to compare with future experimental work.

**Fig. 4:**
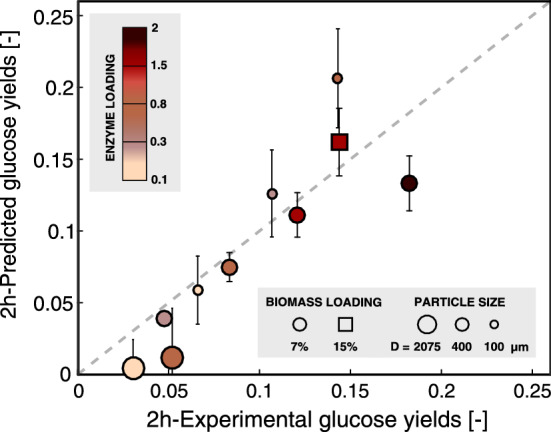
Comparison of predicted initial glucose yields and those measured experimentally from the DS2 dataset for a range of dilute acid-pretreated beech wood substrates (1% SA/160 °C/30 min) (see Additional file 1: A2). Experiments and corresponding simulations were performed over a range of enzyme:initial binding sites molar ratios (0.1–2), biomass loadings (7 and 15%DM), and particle sizes (diameter ranging from 100 to 2075$\mu m$). Standard errors were obtained by propagating the uncertainties associated with pore volume measurements (see Additional file 1: A14)

To illustrate these effects of change in enzyme loading at constant biomass loading, a set of experimental data DS2 was generated on DAP-pretreated beech wood (1% SA/160 °C/30 min) by varying the molar ratio of enzyme:initial binding sites (from $el=0.3$ to $2$ in terms of surface initial coverage, corresponding to $13-85 FPU/g$) for a set biomass loading of 7% dry matter (DM) (Fig. 4). Wet sieving after pretreatment ensured a narrow particle size distribution around $400 \mu m$ and cellulose accessible surface was determined by solute exclusion to be about $24 {m}^{2}/g$ (see “Methods” section). Using the set of parameters *M*_*p*_ = *755* and *τ* = *2* fitted on data set DS1 from the literature [29, 42, 43] (see subsection “The relationship between enzyme loading and cellulose accessibility”), predicted early glucose yields showed good agreement with experimental data DS2, even when working in “enzyme-limited” conditions (Fig. 4). Our ability to accurately capture this kinetic data demonstrated the dependence of the rate on enzyme coverage and the dependence of this coverage on enzyme loading.

However, a larger deviation between predicted and observed glucose yields was observed in the case where a high molar ratio of enzyme:initial binding sites (${e}_{l}=2$) was used to hydrolyze the 400$\mu m$ diameter DAP-pretreated beech wood sample (Fig. 4). This deviation is most likely reflecting the limitations of the assumptions underlying the quantification of both available cellulases and cellulose adsorption sites (i.e. double-slit pore geometry, enzyme surface footprint, homogeneous distribution of component throughout the particle, no distinction between different cellulases constituting the enzyme cocktail) which affected our estimate of minimum enzyme loading required to get the maximal initial rate. In this case, the decrease in predicted yields suggests an underestimation of the number of accessible adsorption sites. Interestingly, the initial number of adsorption sites for all substrates was estimated between $0.12$ and $1.5 \mu mol/g$ of cellulose, which is on the lower end of productive binding measured on cellulosic substrates by Cel7A adsorption ($0.1$ to $10 \mu mol/g)$ [45]. Recent adsorption measurements on lignocellulosic substrates, using two different types of recombinant CMB-proteins on stream exploded pretreated birch/beech wood mixtures, have led to protein coverage of cellulose up to $20 \mu mol/g$ of cellulose [46], with individual coverage by specific CBM-recombinant varying between $5.1\mathrm{~and~} 13.5\,\mu mol/g$ of cellulose. While these lower estimates could rationalize the mismatch observed at higher enzyme loading, other structure- or enzyme-related simplifications could contribute to both over and underestimating the true enzyme loading.

By contrast to variations in enzyme loadings, changes in biomass loadings, where the enzyme-to-biomass ratio is kept constant, showed more limited effects on the course of hydrolysis when considering in silico variations on the substrates featured in dataset DS1 (Fig. 3c). When working at a relatively high molar ratio of enzyme:initial binding sites (${e}_{l}=1$), the reaction was mainly controlled by the rate of enzyme adsorption and desorption with diffusion from bulk only playing a limited role. A higher biomass loading increased the mass transfer rate by accelerating the saturation of the cellulose surface because of the higher enzyme gradient within the particle. However, this phenomenon only marginally improved the rate of glucose release in the first few minutes of hydrolysis (Fig. 3d). In cases where the molar ratio of enzyme:initial binding sites was low (${e}_{l}=0.1$), increasing the concentration gradient by increasing the biomass loading was beneficial at hydrolysis times beyond 5 h (Fig. 3c), which is when the system becomes more diffusion limited after initially being almost entirely reaction-limited due to the low surface coverage. To test the ability of the model to predict early glucose yields upon changes to biomass loadings, enzymatic hydrolysis was performed at a high loading of 15%DM with a molar ratio of enzyme:initial binding sites of 1.5 for the DAP-pretreated beech substrate generated in this study (dataset DS2) (Fig. 4). The model was able to capture the significant increase in glucose titers observed experimentally, confirming the beneficial effect of working at relatively high concentrations of both biomass and enzyme to drive internal mass transfer (Fig. 4). In this case, unaccounted adverse effects related to change in rheological properties [47, 48] and increased enzyme deactivation [31, 48–50] that might have occurred when decreasing amount of free water in the system, did not appear to limit the glucose release. However, such effects are expected to become more pronounced as the biomass loading increases even more, and not taking them into account might further limit a model’s predictive ability.

### Role of particle size reduction on internal mass transfer

**Fig. 5:**
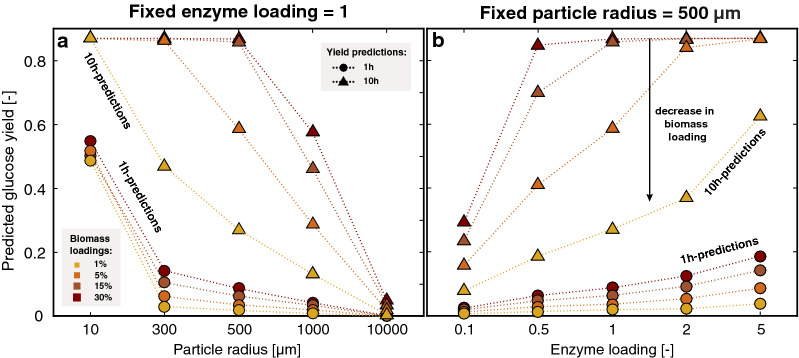
Effect of particle radius and experimental conditions on the predicted yields for a substrate defined in dataset DS1, while varying experimental conditions in silico. Glucose yields after 1 h and 10 h of hydrolysis, for **a** variable particle radius as well as biomass loading at fixed enzyme loading, and **b** fixed particle radius with variable enzyme- and biomass-loading

In this last part, we integrate the role of mass transfer effects as the substrate’s size increases. To illustrate this, we can assume the same model substrate from dataset DS1 (DAP at 220 °C for 7.8 s), but vary the particle radius in silico to assess the effects on glucose release (Fig. 5).

Similar to contradictory results from the literature [32, 47, 48], the important role played by the substrate’s size on the hydrolysis rate depends on the experimental conditions. While increasing enzyme- and biomass-loading favors the enzyme penetration into the substrate by reinforcing the concentration gradient throughout the particle, their effect varies with particle size (Fig. 5a, b). Compared to particles with radius set in silico to an intermediate value, changing biomass loading had limited effects for both small ($R<10 \mu m$) and large ($R\ge 0.1 cm$) particles (Fig. 5a). Increasing solid loading leads to increasing enzyme concentration in the bulk when the enzyme-to-substrate ratio is kept constant. However, for the larger particles, this extra driving force is not sufficient to compensate for increasing internal mass transfer limitations. In such cases, working at enzyme loadings beyond full coverage of adsorption sites helps compensate for limited enzyme penetration into the substrates by maintaining high enzyme concentration in solution and thus limiting any enzyme depletion in the bulk throughout the reaction (Fig. 5b).

These important internal mass transfer limitations could contribute to the lag sometimes observed experimentally in the glucose release as the dry matter loads are increased [48]. In such cases, the progressive liquefaction of the substrate could be reducing large particles to smaller sizes which would transition the overall process from a system that is severely limited by internal mass transfer to one that is less so. This would translate to an accelerating hydrolysis rate.

When compared to experimental results for the newly generated dataset DS2 on DAP-pretreated beech wood (1% SA/160 °C/30 min) ranging from 100 to 2075 μm in radius (see Additional file 1: A2), the model provided reasonable predictions for early glucose yields (Fig. 4). As previously observed, predictions considering higher enzyme loadings exhibit larger deviations from experimental values. However, by contrast to the 400 μm particle discussed above, predictions for the smaller substrate considered (100 μm) at high enzyme loadings overestimated observed yields. In this case, other phenomena, such as deposition of lignin on the cellulose surface or enzyme jamming, may counterbalance the initial underestimation of binding sites.

Overall, even though mismatches occur in the more extreme cases of experimental conditions tested, the strong correlations obtained when predicting early glucose yields over a range of data sets using a single set of fitted parameters validate core modeling assumptions. In this regard, the modeling results can aid in the design of efficient hydrolysis process. For example, while increasing enzyme loading allows rapid degradation of cellulose in relatively small sized substrates, this only works to a point. Loading more cellulases than there are available adsorption sites brings only little benefit. In contrast, for larger size substrates exhibiting important diffusional resistance, high enzyme loadings that are beyond full coverage appear beneficial to increase early glucose release. Tailoring of enzyme quantity to cellulose accessibility is thus not only important for improving process, but also to compare the pretreatment’s ability to promote cellulose degradation. For the latter, since glucose release observed for a given substrate will strongly depend on the enzyme’s ability to completely cover the initial cellulose surface, one should consider comparing systems based on enzyme loadings reported per available surface area instead of per mass of cellulose.

#### Conclusions

We presented a substrate-focused modeling framework based on pore diffusion and surface reaction for the enzymatic hydrolysis of lignocellulosic biomass to evaluate the relative impact of the substrate properties on its digestibility. We demonstrated that the model was robust enough to predict initial glucose release rates for a range of substrates and experimental conditions with a single set of fitted parameters (${M}_{p}=755$ and $\tau =2$), which could be used as a decent first approximation when attempting to predict other substrate–enzyme systems. As such, it constitutes a basis to further investigate the role of particle morphology evolution throughout hydrolysis and later stage kinetics by introducing additional mechanisms, as the evolution of surface accessibility could not solely capture late trend hydrolysis. Implicitly expressing the enzymatic hydrolysis of a cocktail through the use of a single processivity parameter and not accounting for the evolution of the substrate’s surface’s physical properties with time beyond composition was sufficient to reveal the effects of internal mass transfer limitations. However, an improved understanding of the detailed mechanism of the surface–enzyme interactions could help make the model more accurate. In silico results highlighted the importance of mass transfer in controlling cellulose depolymerization when the number of adsorption sites on the cellulose surface outnumbered available cellulases, especially as the substrate size increased. These modeling results highlight the importance of considering multiple process parameters simultaneously and tailoring experimental conditions to the substrate specificities when designing an enzymatic reaction to maximize rates and discussing pretreatment efficiencies. Therefore, modeling can play an important role in designing industrial processes where maximizing rates while minimizing enzyme consumption will play a key economic role.

## Methods

### Model derivation

The particle geometry and several associated assumptions were based on a previously described model, which has been extensively described elsewhere [42, 43] with several improvements. Below, the derivation of the new working equations is presented while summarizing the key hypotheses that were previously described. All symbols are defined and summarized in Table 1. Briefly, biomass fragments are modeled as a non-shrinking, porous cylinder of radius $R$ containing uniformly distributed lignin, hemicellulose and cellulose, with a defined fraction of its volume being susceptible to hydrolysis (Fig. 1) [42, 43]. In addition to assumptions made for the particle morphology, a stepwise model for the enzymatic degradation is used: the enzyme that has reached the enzyme surface through pore diffusion, can adsorb, react and desorb (Fig. 1). Pore diffusion refers to internal mass transfer that, based on initial calculations, is much more significant than external mass transfer, which is ignored in this work (see Additional file 1: A4). For convenience, we account for the loss of soluble cellulose oligomers at the desorption step. From the substrate’s perspective, the enzymatic process translates as a change in biomass porosity. Importantly, two significant improvements are made over the previous model: (1) we now include the effect of enzyme depletion in the bulk solution, which, as we will show, can be influential at lower enzyme loadings and for larger particles; and (2), we include the effect of enzyme dilution caused by the increase in porosity. Interestingly, the latter inclusion only has a significant effect for cases where the former inclusion has a significant effect, which demonstrates the importance of coupling these two effects, and of their inclusion for studying low enzyme loadings. Finally, the consequences of products released in solution, such as inhibition or crowding within pores, as well as the action of specific enzymes and mechanisms of action, are not accounted for.

Within this framework, the set of equations describing the simultaneous diffusion and reaction of enzymes in the accessible portion of the particle is obtained by performing a mole balance over a thin cylindrical segment $\Omega =\left\{\left(r,\varphi , z\right) \in {\mathbb{R}}^{3}:r\le r\le r+dr, 0\le \varphi \le 2\pi , 0\le z\le {L}_{A}\right\}$ of volume $dV(r)$, which contains both the biomass and the pores. The concentration of enzymes in the pores ${C}_{E}^{F}\left(r,t\right)$ in this domain is:

1 $$\frac{d}{dt}\left[\varepsilon \left(r,t\right){C}_{E}^{F}\left(r,t\right)\right],$$

where $\varepsilon (r,t)$ is the porosity. Three mechanisms affect the time evolution of the enzyme’s fluid concentration: the diffusion into the particle, the adsorption–desorption process from the cellulose surface, and the dilution of enzymes within pores due to more void being created during hydrolysis. All these effects are described mathematically below.

First, using Fick’s first law and assuming that the radius $R$ of the particle is much smaller than its length ${L}_{A}$, which allows us to ignore end effects, the diffusion contribution to the enzyme balance over a cylinder slice is expressed as:

2 $${\nabla }_{r}\left[\varepsilon \left(r,t\right){D}_{E}{\nabla }_{r}{C}_{E}^{F}(r,t)\right]={\mathrm{D}}_{E}\left[\frac{{\partial }^{2}{C}_{E}^{F}(r,t)}{{\partial r}^{2}}+\frac{1}{r}\frac{\partial {C}_{E}^{F}(r,t)}{\partial r}+\frac{1}{\varepsilon (r,t)}\frac{\partial \varepsilon (r,t)}{\partial r}\frac{\partial {C}_{E}^{F}(r,t)}{\partial r}\right],$$

where the right-hand side of Eq. (2) is the development of the derivative on the left. In this equation, ${D}_{E}$ is the time-independent part of the effective diffusivity ${D}_{E}^{eff}\left(r,t\right)$ parameter that is based on the average pore diffusivity ${\stackrel{-}{D}}_{E}^{pore}$ and tortuosity $\tau$ of the pore network as well as the evolving porosity $\varepsilon (r,t)$ [51],

3 $${D}_{E}^{eff}\left(r,t\right)=\frac{{\varepsilon (r,t)\stackrel{-}{D}}_{E}^{pore}}{\tau }=\varepsilon \left(r,t\right){D}_{E}.$$

The removal of enzymes from solution through adsorption can be accounted for by including the term describing the change in enzyme surface concentration $\left(\frac{\partial {C}_{E}^{S}\left(r,t\right)}{\partial t}\right)$. With all these terms combined, the mass balance of free enzymes in solution becomes:

4 $$\frac{\partial {C}_{E}^{F}(r,t)}{\partial t}={D}_{E}\left[\frac{{\partial }^{2}{C}_{E}^{F}(r,t)}{{\partial r}^{2}}+\frac{1}{r}\frac{\partial {C}_{E}^{F}(r,t)}{\partial r}+\frac{1}{\varepsilon (r,t)}\frac{\partial \varepsilon (r,t)}{\partial r}\frac{\partial {C}_{E}^{F}(r,t)}{\partial r}\right]-\frac{\partial {C}_{E}^{S}\left(r,t\right)}{\partial t}-\frac{{C}_{E}^{F}\left(r,t\right)}{\varepsilon \left(r,t\right)}\frac{\partial \varepsilon \left(r,t\right)}{\partial t}.$$

In Eq. (4), the accumulation of free enzymes in solution is equated to a diffusion term, an adsorption term and a dilution term arising from the rearrangement of Eq. (1). Both the time evolution of porosity and bound enzymes remain to be defined. The latter is described by a time-dependent Langmuir isotherm, with the concentration of enzymes at the cellulose surface given by:

5 $$\frac{\partial {C}_{E}^{S}\left(r,t\right)}{\partial t}={k}_{ads}{C}_{E}^{F}\left(r,t\right)\left[{C}_{E,max}^{S}\left(r,t\right)-{C}_{E}^{S}\left(r,t\right)\right]-{k}_{des}{C}_{E}^{S}\left(r,t\right),$$

where ${k}_{i}$ designates the adsorption ($i=ads$) or desorption ($i=des$) rate constant and ${C}_{E,max}^{S}\left(r,t\right)$ is the maximum concentration of enzymes bound to the surface at a given time $t$ (see Additional file 1: A5). Within this formulation, no complexation/decomplexation steps are directly represented. Values for both adsorption and desorption rate constants were found in the literature [42, 43, 60] and fell within ranges of other reported estimates (see Additional file 1: A6). In addition, sensitivity analyses were carried out on both rate constants, as well as the enzyme diffusivity parameter and combined fitted parameter M_p_ and $\tau$ (see Additional file 1: A7). Results show that, when keeping M_p_ constant, changes in the desorption rate constant strongly impact glucose release for cases where hydrolysis is kinetically limited (i.e. for substrates that are not diffusion limited due to low cellulose accessibility). In such systems, the glucose release depends almost exclusively on the reaction rate, and more particularly the catalytic rate constant, that we show to be equivalent to the combination of M_p_ and the desorption rate constant in our formulation (see Eq. A.4 in Additional file1: A1). In contrast, glucose release is less sensitive to the kinetic parameters for systems where the internal diffusion is significantly impacted by the particle physical properties. The total number of adsorption sites is defined as the available cellulose surface multiplied by a parameter $\sigma$, which defines the moles of enzyme binding sites per surface unit. The latter is determined based on geometrical considerations, assuming a spherical footprint with a diameter of $51 \AA$ for the cellulase on the surface.

As the reaction proceeds and the accessible cellulose is hydrolyzed, the available surface for enzyme binding is assumed to gradually decrease together with the cellulose content. As the decrease of cellulose content is directly proportional to the increase in porosity (i.e. the dissolution of cellulose into soluble sugars during the enzymatic hydrolysis increases the internal volume), the available number of binding sites on the cellulose surface decreases linearly with the increase in porosity as cellulose is consumed. While potential particle fragmentation and particle swelling resulting from enzymatic action might change the available cellulose surface, both of these mechanisms are assumed to have minor effect on the available cellulose surface at early hydrolysis times compared to the surface erosion mechanism considered here. Based on the infinite slit model for biomass pores, the initial cellulose surface area is computed from pore volumes assuming that only two flat pore wall areas contribute to the overall accessible surface for pores larger than twice the diameter of a cellulase and that only one wall contributes to the surface area for pores with widths between one and two cellulase diameters [42, 43] (see Additional file 1: Figure S7 in A8).

Since cellulose hydrolysis is accounted for as enzymes detach from the surface, the time evolution of the porosity, which tracks the progression of hydrolysis, can be equated to the rate of enzyme desorption:

6 $$\frac{\partial \varepsilon \left(r,t\right)}{\partial t}=\frac{{{k}_{des}C}_{E}^{S}\left(r,t\right){M}_{p}{MM}_{glu}{H}_{glu}}{{\rho }_{C}^{IV}},$$

where ${M}_{p}$ represents the moles of glucose released in solution per binding cycle and per mole of enzyme. Here, the term is expressed as mass of cellulose degraded per binding cycle per mole of enzyme through the hydrolysis factor ${H}_{glu}$ and molar mass of glucose ${MM}_{glu}$, and ${\rho }_{C}^{IV}$ is the total cellulose density (accounting for both accessible and inaccessible pore volume, i.e. with diameter smaller than one of a cellulase). The system of coupled partial differential equations (PDEs) composed of Eqs. (4), (5) and (6) describing the diffusion of enzymes inside a porous biomass particle and its subsequent hydrolysis can then be solved if defined boundary (BC) and initial (IC) conditions are provided. Assuming that all enzymes are initially contained in the bulk solution ${V}_{B}$, we have

7 $$\text{IC }\left\{\begin{array}{c}{C}_{E}^{F}\left(r,0\right)=0~~ \forall ~ r\ne R\\ {C}_{E}^{s}\left(r,0\right)=0~~ \forall ~r\\ \varepsilon \left(r,0\right)={\varepsilon }_{0}~~ \forall ~ r\ne R\end{array}\right. \begin{array}{c}~~~{C}_{E}^{F}\left(R,0\right)={C}_{E,0}^{F}={n}_{E,0}^{F}/{V}_{B}\\ \\ \varepsilon \left(R,0\right)=1.\end{array}$$

The depletion of enzymes in the bulk solution is here accounted for by integrating the flux of enzymes passing through the biomass particle external surface ${S}_{cyl}$ (see Additional file 1: A9). Coupled to a no-flux boundary condition at the center of the particle, as longitudinal diffusion is neglected, the boundaries conditions are expressed as:

8 $$\text{BC }\left\{\begin{array}{c}{\left.\frac{\partial {C}_{E}^{F}(r,t)}{\partial t}\right|}_{r=R}=-\frac{{S}_{cyl}}{{V}_{Bulk}}\varepsilon (R,t){D}_{E}{\left.\frac{\partial {C}_{E}^{F}(r,t)}{\partial r}\right|}_{r=R}\\ {\left.\frac{\partial {C}_{E}^{F}(r,t)}{\partial t}\right|}_{r=0}=0\\ {\left.\frac{\partial \varepsilon \left(r,t\right)}{\partial t}\right|}_{r=0}=0.\end{array}\right.$$

Here, any external mass transfer phenomena are disregarded. Also, because the external surface usually represents only a small fraction of the accessible cellulose surface area, and because it is difficult to measure, enzyme adsorption on the external surface of the biomass particle is neglected (see Additional file 1: A10).

Parameters extracted from literature include: adsorption and desorption rate constants and cellulase diffusivity in bulk solution. Parameters fitted to the data include: tortuosity and average mass of cellulose liberated per binding cycle. The remaining variables are calculated based on available experimental data, including: the measured initial accessible pore volume and cellulose fraction, used to compute initial cellulose surface area, and final glucose yield, used to predict the amount of cellulose that can be hydrolyzed.

The enzyme ${e}_{l}$- and biomass ${b}_{l}$-loading are two key experimental parameters, and are used to define the initial enzyme bulk concentration:

9 $${\mathrm{C}}_{\mathrm{E},0}^{\mathrm{F}}=\frac{{\mathrm{n}}_{\mathrm{E},0}^{\mathrm{F}}}{{\mathrm{V}}_{\mathrm{B}}}=\frac{{\mathrm{e}}_{\mathrm{l}}{\int }_{0}^{R}{\mathrm{S}}_{\mathrm{C}}(r,t=0)dr\upsigma }{{\mathrm{b}}_{\mathrm{l}}}.$$

Here, the enzyme loading is expressed in terms of the initial moles of adsorption sites per mass of substrate, i.e. an enzyme loading of 2 corresponds to twice the amount of enzymes required to cover all initially accessible binding sites on the cellulose surface. The calculation results are used to chart the time-course of enzymatic hydrolysis.

### Numerical implementation

Numerical solutions for the system of PDEs are obtained using the Method of Lines [52], in which all but one variable are discretized, leading to a system of decoupled ordinary differential equations (ODEs) for which efficient solvers exist. Here, the spatial coordinate representing the particle radius is partitioned into $n$ regions, with layer $n+1$ representing the surrounding bulk solution. The resulting $3(n+1$) coupled ODEs, mirroring the initial PDEs system, are then solved in a dimensionless form (see Additional file 1: A5) using the ODE solver *ode15s* [53] in Matlab [54]. The correctness of the implemented algorithm was evaluated by ensuring conservation of enzymes (within < 2% of the initial loading) over the time-course of the simulation (see Additional file 1: Figure S8 in A11).

### Pretreated beech wood

**Fig. 6:**
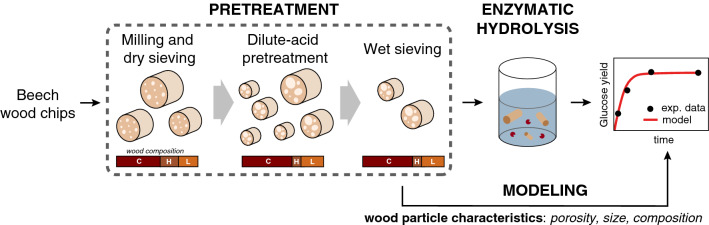
Schematic representation of the experimental process. In brief, wood chips were milled and dry sieved before undergoing a dilute acid pretreatment affecting both their composition (here schematically indicated as C/H/L for cellulose, hemicellulose (measured as the xylan content) and lignin, respectively) and physical characteristics. Wet sieving after pretreatment ensured a narrow particle size distribution. The resulting wood sample was then subjected to enzymatic hydrolysis and glucose release was measured. Modeling of the reaction was based on experimentally measured physical characteristics of the particle; specifically, the pore size distribution, particle size distribution, and the composition

Air-dried beech wood (*Fagus sylvatica*) chips collected from Zollikofen (Switzerland) were first milled to pass through a 2-mm screen. These particles were further sieved and those between $250$ and $450 \mu m$ in diameter were retained as the so-called native substrate. This substrate was further processed using dilute acid—1wt% sulfuric acid (SA/Merck, 100,732) at 160 °C—in 60 ml glass reactors at a loading of 2 g of dry substrate per 20 ml acid solution for 30 min, followed by Büchner filtration and extensive washing with purified water (Milli-Q grade). To allow fiber swelling, wood particles were pre-soaked overnight at 4 °C in the pretreatment solution. Wet pretreated wood sample was wet sieved ($300-500 \mu m$ diameter) under purified water (Milli-Q grade) and then kept for a maximum of 2 weeks in sealed plastic bags at 4 °C prior to further utilization, to avoid drying and degradation. A schematic representation of the experimental process is summarized in Fig. 6.

Composition analysis of the pretreated wood sample was performed according to the LAP procedure published by the National Renewable Energy Laboratory [55] (see Additional file 1: A12).

### Enzymatic hydrolysis

Enzymatic hydrolysis was carried out in a citrate buffer (pH = 4.8/Sigma, C1909, 71,402) as described previously [56] using a commercial enzyme blend (150 FPU/g, Cellic CTec2, Novozyme, Denmark/Sigma, SAE0020) at various molar ratio of enzyme:initial binding sites corresponding to ratios of 0.1–2. In addition, tetracycline (Sigma, 87,128) and cycloheximide (Sigma, C7698) were added to the reaction medium to avoid undesired bacterial and fungal growth, respectively. Protein content was assayed to 55.1 mg protein/ml according to the Bradford method [57] using the commercially available Pierce Coomassie protein assay kit (ThermoFisher, 23,200).

### Pore size distribution

To avoid any change in porosity that might occur due to hornification during drying, pore size distribution was determined in wet conditions using a modified batch solute exclusion technique [58, 59]. A series of suitably sized PEGs as well as glucose (Sigma, G8270) were used as molecular probes (see Additional file 1: Table S3 in A13). Wet wood samples (${m}_{wood,wet}=0.4 g$) were incubated with the probe solution in ultrapure water (${V}_{probe}=0.35 ml,$ ${C}_{probe,init}=50 g/L$) for 3 h with occasional mixing, followed by which, the supernatant was recovered by centrifugation (2500 rpm or ~ 1000*g*, 15 min) through $3 \mu m$ centrifugal filters. The resulting solution was further diluted with Milli-Q water and the final probe concentration ${C}_{probe, final}$ was measured using a refractive index detector (Viscotek VE 3580) connected to a syringe pump—with an injection volume of 3 ml at 0.5 ml/min at a detection temperature of 35 °C. The remaining solids were then washed and dried to record the dry weight and moisture content ${x}_{wat}$. For each set of recorded data, the refractive index of a blank solution obtained by incubation of the wood samples with Milli-Q water was used to correct the signal for any soluble material that could interfere with quantification before computing pore volumes (see Additional file 1: A13). Prior to any measurements on the native substrate, the particles were soaked for at least 48 h to allow fiber swelling, with daily water changes to avoid bacterial/fungal contamination.

## Supplementary Information

**Additional file 1:** Supplementary document providing additional figures, tables and relevant derivations.

## Data Availability

The datasets used and/or analyzed during the current study are available from the corresponding author on reasonable request.

## Abbreviations

BC: Boundary conditions
DAP: Dilute acid pretreatment
DM: Dry matter
IC: Initial conditions
ODE: Ordinary differential equation
PDE: Partial differential equation
SA: Sulfuric acid
