# Supplementary material for: Investigating the effects of substrate morphology and experimental conditions on the enzymatic hydrolysis of lignocellulosic biomass through modeling
Source: Biotechnol Biofuels. 2021 Apr 26;14:103. doi: 10.1186/s13068-021-01920-2 (PMC8073973; doi:10.1186/s13068-021-01920-2)
Supplement: Supplementary file 1 — Additional file 1: Supplementary document providing additional figures, tables and relevant derivations. [file 13068_2021_1920_MOESM1_ESM.pdf]

SUPPLEMENTARY INFORMATION for:

Investigating the effects of substrate morphology and experimental conditions on the enzymatic hydrolysis of lignocellulosic biomass through modeling

Jessica C. Rohrbach[1], Jeremy S. Luterbacher[1]*

[1]Laboratory of Sustainable and Catalytic Processing, Institute of Chemical Sciences and Engineering, École Polytechnique Fédérale de Lausanne (EPFL), CH-1015 Lausanne, Switzerland; e-mail: jeremy.luterbacher@epfl.ch

A0. Particle Shape

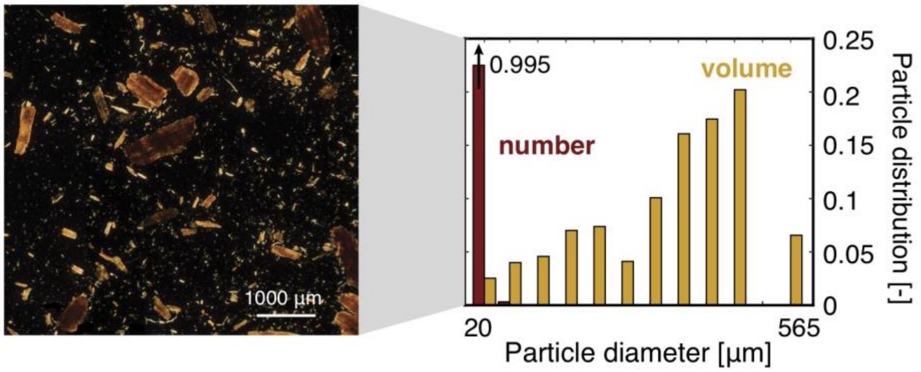

Figure S0. Particle size distribution and representative image of DAP pretreated beech wood (1%SA / 160°C / 30min) from native particle presenting diameter between 250-450 um. Both distribution and image represent the pretreated substrate before wet sieving. These images show that the approximation of biomass particles as long cylindrical particles is reasonably accurate and closer than most other simple geometries.

A1. Comparison M_p with processivity

In our model formulation, the release of glucose in solution resulting from the hydrolytic action of cellulases is expressed as a change in particle porosity ε(*r*,*t*). Within the model framework, such glucose release is accounted for as the enzyme desorbs from the

surface. Mathematically, change in the particle porosity can thus be expressed as:

$$\frac{\partial \varepsilon(r,t)}{\partial t} = \frac{k_{des} C_E^S(r,t) M_p MM_{glu} H_{glu}}{\rho_C^{IV}} \quad (A.1)$$

where $k_{des} C_E^S(r,t)$ is the enzyme desorption rate from the cellulose surface, M_p is an adimensional parameter representing the number of glucan unit released per enzyme desorbing from the surface (i.e. mole of glucose released per mole of enzymes desorbing), $MM_{glu}$are the molar mass and hydrolysis factor of glucose respectively, and ρ_C^{IV} is the density of cellulose including the pore volume, which varies with porosity. These two constants and variable are essentially responsible for translating the mass of glucose released into a pore volume that is created as a result of cellulose hydrolysis and this resulting glucose release. By dividing both side of Eq. (A.1) by these three terms, we can reformulate this equation describing a change in porosity into an equation describing a change of glucose concentration:

$$\frac{\rho_C^{IV}}{MM_{glu} H_{glu}} \frac{\partial \varepsilon(r,t)}{\partial t} = \frac{\partial}{\partial t}\left[\frac{\varepsilon(r,t)\rho_C^{IV}}{MM_{glu} H_{glu}}\right] = \frac{\partial C_{glu}(r,t)}{\partial t} = k_{des} C_E^S(r,t) M_p \quad (A.2)$$

This translation allows us to compare our model more directly to a general and common mechanistic model of cellulose hydrolysis by single enzymes (Nill et al., 2018), where the glucose release is dictated by the processive action of productively bound cellulases hydrolysing cellulose into glucose at a rate given by the catalytic rate constant k_{cat} [Figure S0]:

$$\frac{\partial C_{glu}(r,t)}{\partial t} = k_{cat} C_E^S(r,t) \quad (A.3)$$

Comparing the common mechanistic model (Eq. (A.3)) and our transformed equation model (Eq. (A.2)), we obtain the following equivalence:

$$k_{cat} = k_{des} M_p \quad (A.4)$$

On an ideal cellulose polymer (i.e. where the catalytic action of an adsorbed cellulase is independent of its location on the cellulose surface), the number of catalytic events n_{int} that a cellulase can perform before desorbing from the surface (i.e. the so-called intrinsic processivity), is related to both catalytic and desorption constant (Kurasin et al., 2011):

$$n_{int} = \frac{k_{cat} + k_{des}}{k_{des}} \quad (A.5)$$

Combining Eqs. (A.5) and (A.4), we obtain:

$$k_{cat} = k_{des}(n_{int} - 1) = k_{des}M_p \quad (A.6)$$

leading to the following equivalence:

$$M_p = n_{int} - 1 \quad (A.6)$$

Considering that values of intrinsic processivity for crystalline cellulose fall in the range of ~4000, M_p and n_{int} are essentially equal (Kurasin et al., 2011). Therefore, M_p should be understood as an estimate of the average intrinsic processivity per enzyme for a mixed enzyme cocktail. Because the definitions differ slightly and because intrinsic processivity is defined in the literature for a given enzyme as opposed to a mixture, we used a different designation in this work. Nevertheless, mathematically, n_{int} and M_p are essentially equivalent in both model formulations. The estimated values of M_p in this study lie between typical values for apparent (~50) and intrinsic (~4000) processivity for an exoglucanase on a crystalline cellulose surface. This observation can be explained by the fact that we are (i) considering the action of an enzyme cocktail and not a single enzyme and (ii) dealing with a lignocellulose surface that is far from an ideal polymer. In addition, the computed number of adsorption sites for the substrates considered in this study lies in the range of what can be found in literature for similar substrates.

A2. Model – experimental inputs

Table S1 shows an overview of the available data found in literature for the various substrates used as inputs for the model in this work (Grethlein, 1985).

SUBSTRATE	GLUCOSE YIELD [-]		Cellulose fraction [-]	Accessible surface [m^2/g]
	2h	24h		
Native (90% Birch/10% Maple)	0.0410	0.1530	0.42	6.3
DA-1%sulfuric acid/100°C/5hr	0.1470	0.2180	0.55	21.6
DA-1% sulfuric acid/180°C/7.8s	0.2430	0.4260	0.60	23
DA-1% sulfuric acid/200°C/7.8s	0.4180	0.8540	0.67	59
DA-1% sulfuric acid/220°C/7.8s	0.64	0.87	0.62	80.9

Table S1. Characteristics of the various substrates used in this study (Grethlein, 1985; Luterbacher et al., 2013)

The overall digestibility, defining the accessible cellulose fraction, is taken as the reported 24h-glucose yields. Particle radii are assumed to correspond to the average size between sieves used to screen the native biomass particles (i.e. $R = 25\ \mu m$). Enzymatic hydrolysis was carried out at a low solid loadings of 2% in citrate buffer pH=4.8 at 50°C for 24h, using cellulase powder from the Trichoderma Reesei Rutger's C strain (92.5mg/100ml) complemented by a source of β-glucosidase (0.1ml).

In addition to these data, a new data set was generated from beech wood, the characteristics of which are summarized in Table S2. Beech wood particles were here subjected to dilute sulfuric acid pretreatment in typical lab-scale conditions (Shi et al, 2011, Sun et al. 2014). The final glucose yield was assumed to be that reached after 120h when the enzymatic hydrolysis was performed at high enzyme loadings to avoid incomplete cellulose degradation stemming from enzyme-related limitations. In any case, sensitivity analysis performed to evaluate this influence of change in the final yields on the predictions of the 2h-glucose yields showed only a marginal effect [Figure S3]. The recorded concentration of cellobiose throughout hydrolysis was marginal in comparison to the glucose concentrations, as illustrated in Figure S4.

Substrate diameter [lower limit, upper limit] [µm]	Final glucose yield - 120h [-]	Cellulose fraction [-]	Accessible surface [m^2/g]
[50,150]	0.8380	0.61	35.8 ± 5.1
[300,500]	0.6330	0.62	24.7 ± 3.6
[1000,3150]	0.6773	0.61	33.9 ± 3.1

Table S2. Characteristics of the substrates generated in this study

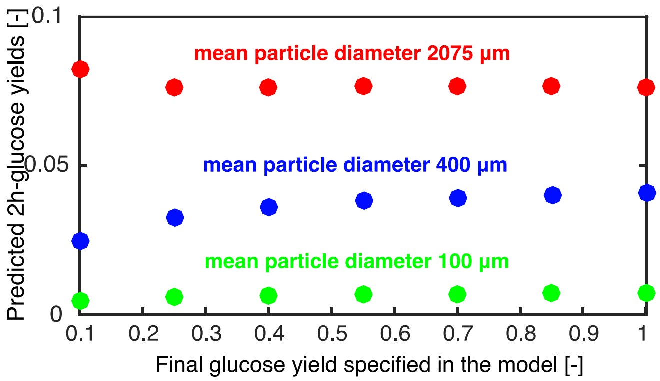

Figure S1. Predicted 2h-glucose yields as a function of the specified final glucose yield in the model for pretreated beech wood (1%SA/160°C/30min) with different particle sizes. Simulations were performed for a low enzyme loading of 0.3.

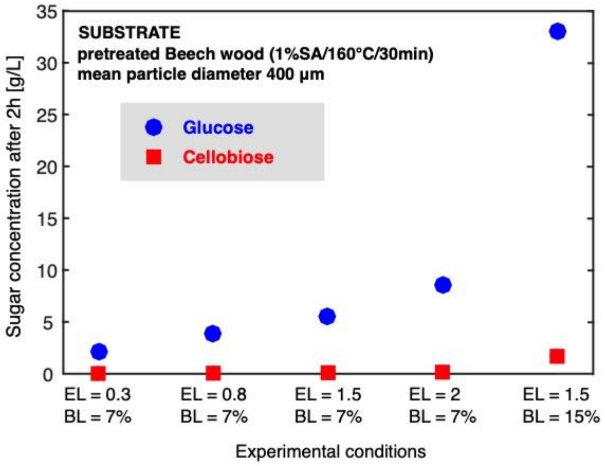

Figure S2. Experimental glucose and cellobiose concentrations measured after 2h of hydrolysis for pretreated beech wood (1%SA/160°C/30min) for several enzyme and biomass-loadings.

Experimental enzyme loadings were expressed as normalized to the number of initially accessible binding sites on the cellulose surface assuming an average molecular mass of

51.8 kDa for the cellulases making up the enzyme cocktail used experimentally (Seiboth et al., 2011).

A3. Individual and combined optimization of M_p and τ

Tortuosity values were rationally bound between 1 and 7, which represent situations from the case where all pores are aligned with the diffusive flux to extreme cases of tortuosity (Davis et al., 2003). When fitting M_p over the whole set of data, only marginal differences in fit were obtained for values of tortuosity up to 4-5 [Figure S5], with slight decreases in goodness-of-fit when increasing the diffusion resistance. The choice of the optimal parameter to use for simulations was then purely based on the slightly improved R^2 value (leading to the optimal value of $M_p = 755$ and $\tau=2$). When instead performing individual fits for each substrate [Figure S6], we did not observe any clear trend based on substrate characteristics (e.g. pretreatment severity), indicating again that the dependence on accessibility dominated the kinetics and that small changes in Mp or τ are likely due to uncertainty in parameter estimation rather than the result of physical phenomena.

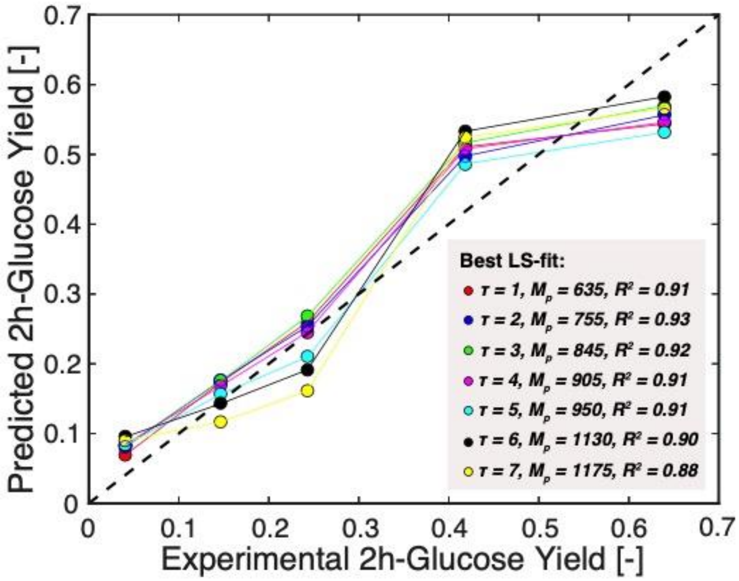

Figure S3. Optimal value of $\mathbf{M_p}$ in terms of least-square fitting for each $\boldsymbol{\tau}$ considered to predict early glucose yields, with indication of the goodness-of-fit parameter $\boldsymbol{R^2}$. Fits were performed on the whole data set DS1. Predictions were performed for the mixed hardwood substrates considered in the study (Grethlein, 1965).

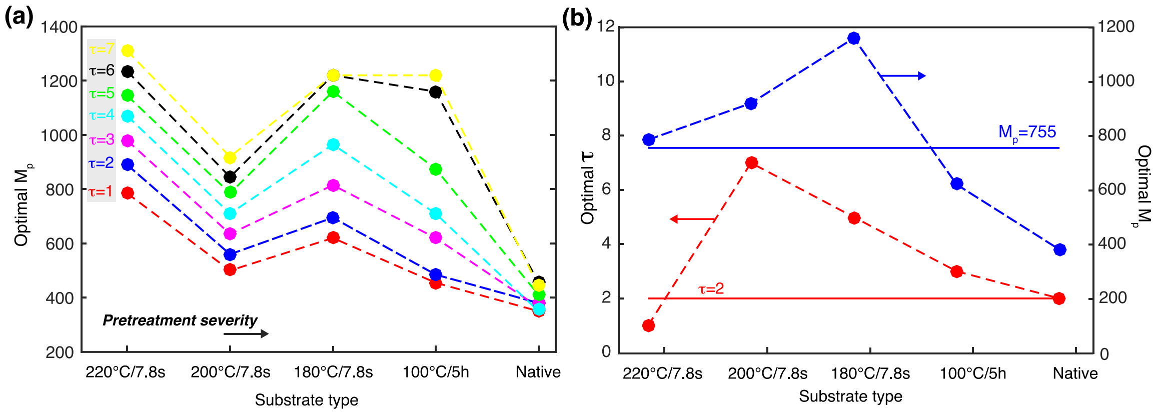

Figure S4. (a) Optimal values of $\mathbf{M_p}$ for a range of $\boldsymbol{\tau}$ after least-square fitting to predict early glucose yields for different pretreatment conditions. (b) Combined optimal values of $\mathbf{M_p}$ and $\boldsymbol{\tau}$ after least-square fitting to predict early glucose yields for different pretreatment conditions. For comparison, the values of these parameters (M_p = *755* and <u>*τ=2*</u>) on which calculations are based in this study are shown with the horizontal lines. In both cases, fits were performed on each substrate individually. Predictions were performed for mixed hardwood substrates considered in the study (dataset DS1) (Grethlein, 1985).

A4. Effect of external mass transfer

The potential impact of external mass transfer on the overall reaction rate of cellulose depolymerization was evaluated using the modified Weisz-Prater criterion for external mass transfer (Davis et al., 2003),

$$\frac{r_{obs} R_p}{\bar{k}_c C_{E,bulk}} < \frac{0.15}{n},$$

where r_{obs} is the observed reaction rate, R_p the particle radius, $C_{E,bulk}$ the enzyme bulk concentration, n is the reaction order and $\bar{k}_c$ the mass transfer coefficient. Satisfying this relationship indicates that internal mass transfer or reaction controls the reaction and that external mass transfer can be neglected.

The mass transfer coefficient $\bar{k}_c$ can be estimated from a correlation dependent on both the Reynolds Re and Schmidt Sc numbers

$$Re = \frac{\bar{\mu}}{\rho D_E} \qquad and \qquad Sc = \frac{u\rho L}{\bar{\mu}},$$

through the Sherwood number Sh,

$$Sh = \frac{\bar{k}_c L}{D_E} = 2 + 0.6 Re^{1/2} Sc^{1/3},$$

leading to

$$\bar{k}_c = \frac{2D_E}{L} + \frac{0.6 D_E^{2/3} u^{1/2} \rho^{1/6}}{L^{1/2} \bar{\mu}^{1/6}}.$$

While the viscosity $\bar{\mu}$ and density ρ of the sodium citrate buffer containing the enzymes is assumed to be same as those for water, the fluid velocity is calculated based on the flask internal radius ($R_{flask} = 1.25 \cdot 10^{-2}\ m$) and incubator rotation speed ($\omega_{incubator} = 120\ rpm$),

$$u = \frac{2\pi R_{flask}\omega}{60},$$

and the particle characteristic length L is assumed to be $2R_p$. As a first approximation, we assumed that the observed rate corresponded was linked to the average internal rate of reaction, i.e. the rate of cellulose depolymerization $r_{dep,cellulose}$, which is ultimately what the model predicts:

$$r_{obs} \approx -\frac{1}{M_p} r_{dep,cellulose} = \frac{1}{M_p} \frac{dC_{glucose,released}}{dt}.$$

Since we are comparing enzyme mass transfer to its participation in the surface reaction, the quantity of glucose generated per amount of time is divided by the factor M_p to calculate the quantity of enzyme participating in the reaction per time. This factor accounts for the fact that several glucose molecules are generated for one enzyme going through a binding cycle.

The Weisz-Prater criterion was evaluated for the initial predicted reaction rate (the fastest rate, and thus the most likely to be controlled by external transfer) for two extreme particle sizes,

$$R_p = 10\ \mu m: \qquad \frac{r_{obs} R_p}{\bar{k}_c C_{E,bulk}} = 9.4 \cdot 10^{-7} < 0.15,$$

$$R_p = 1\ cm: \qquad \frac{r_{obs} R_p}{\bar{k}_c C_{E,bulk}} = 2.4 \cdot 10^{-4} < 0.15.$$

In both cases, predicted initial rates with respect to enzyme concentration, closely followed a first order reaction rate, thus $n = 1$ was used as a first approximation. Altogether, the values were far below the criteria requirement. Therefore, even though the estimation of r_{obs} and $\bar{k}_c$ were based on several approximations, we can safely assume that external mass transfer plays a negligible role on enzymatic hydrolysis and can safely be ignored.

A5. Working equations – surface enzyme concentration and non-dimensional formulation

The time evolution of the maximum enzyme concentration at the cellulose surface $C_{E,max}^S(r,t)$ can be expressed as function of the porosity $\varepsilon(r,t)$:

$$C_{E,max}^S(r,t) = \frac{\sigma S_c(r,t)}{V_p(r,t)} = \frac{\sigma S_{c,0}}{V_{p,0}} \cdot \frac{\varepsilon_0}{\varepsilon(r,t)} \cdot \frac{\varepsilon_\infty - \varepsilon(r,t)}{\varepsilon_\infty - \varepsilon_0}$$

where σ is the parameter representing the number of moles of enzymes per unit of surface, $V_{p,0}$ is the initial accessible pore volume and $S_{c,0}$ the initial cellulose surface.

Here, we assumed that the available surface for adsorption tends to 0 as the cellulose hydrolysis goes to completion, i.e.:

$$S_c(r,t) = S_{c,0}(r,0)\frac{\varepsilon_\infty - \varepsilon(r,t)}{\varepsilon_\infty - \varepsilon_0}$$

where ε_0 and ε_∞ corresponds respectively to the initial and final porosity (which occurs when the defined accessible cellulose is completely degraded).

Prior to being solved, the surface- and fluid-concentration of enzymes ($C_E^S(r,t)$ and $C_E^F(r,t)$, respectively) are scaled with the initial bulk concentration $C_{E,0}$.

$$C_E^F(r,t) \longrightarrow \chi_E^F(r,t) = \frac{C_E^F(r,t)}{C_{E,0}} \qquad and \qquad C_E^S(r,t) \longrightarrow \chi_E^S(r,t) = \frac{C_E^S(r,t)}{C_{E,0}},$$

These variables are introduces into the dimensional working equations defined by Eqs. (4)-(5) and (6), resulting in an equivalent partially non-dimensional system of coupled equations,

$$\begin{cases} \dfrac{\partial \chi_E^F(r,t)}{\partial t} = D_E\left[\dfrac{\partial^2 \chi_E^F(r,t)}{\partial r^2} + \dfrac{1}{r}\dfrac{\partial \chi_E^F(r,t)}{\partial r} + \dfrac{1}{\varepsilon(r,t)}\dfrac{\partial \varepsilon(r,t)}{\partial r}\dfrac{\partial \chi_E^F(r,t)}{\partial r}\right] \\ \qquad\qquad - \dfrac{1}{\varepsilon(r,t)}\dfrac{\partial \chi_E^S(r,t)}{\partial t} - \dfrac{\chi_E^F(r,t)}{\varepsilon(r,t)}\dfrac{\partial \varepsilon(r,t)}{\partial t} \\ \dfrac{\partial \chi_E^S(r,t)}{\partial t} = \dfrac{\partial}{\partial t}\left\{k_{ads}\chi_E^F(r,t)C_{E,0}\left[\chi_{E,max}^S(r,t) - \chi_E^S(r,t)\right] - k_{des}\chi_E^S(r,t)\right\} \\ \dfrac{\partial \varepsilon(r,t)}{\partial t} = \dfrac{k_{des}\chi_E^S(r,t)C_{E,0}M_p}{\rho_C^{IV}}, \end{cases}$$

with associated BC and IC,

$$\text{BC}\begin{cases} \left.\dfrac{\partial \chi_E^F(r,t)}{\partial t}\right|_{r=R} = -\dfrac{S_{cyl}}{V_{Bulk}}\varepsilon(R,t)D_E\left.\dfrac{\partial \chi_E^F(r,t)}{\partial r}\right|_{r=R} \\ \left.\dfrac{\partial \chi_E^F(r,t)}{\partial t}\right|_{r=0} = 0 \\ \left.\dfrac{\partial \varepsilon(r,t)}{\partial t}\right|_{r=0} = 0, \end{cases}$$

$$\text{IC} \begin{cases} \chi_E^F(r,0) = 0 \quad \forall\, r \neq R \quad \chi_E^F(R,0) = 1 \\ \chi_E^S(r,0) = 0 \qquad \forall\, r \\ \varepsilon(r,0) = \varepsilon_0 \quad \forall\, r \neq R \quad \varepsilon(R,0) = 1. \end{cases}$$

The discretization of the radial dimension into n sub-regions $\omega_i = \{(r,\ \varphi, z) \in \mathbb{R}^3, i \in [1, n]\colon\ (i-1)\,{}^{R}/_{n} \leq r \leq i\,{}^{R}/_{n},\ 0 \leq \varphi \leq 2\pi,\ 0 \leq z \leq\ L_A\}$ as proposed by the method of lines is then based on the central finite difference, where the first $f'(x)$ and second $f''(x)$ derivatives are approximated as:

$$f'(x) \approx \frac{f(x+h) - f(x-h)}{2h},$$

$$f''(x) \approx \frac{f(x+h) - 2f(x) + f(x-h)}{h^2}$$

where h is the size of the discretized step and $f(x)$ is the function of interest.

A6. Model parameters – rate constants

In this work, we assumed a general desorption rate constant, with no distinction made between desorption and decomplexation rate. This global desorption rate constant used was $k_{des} = 0.011\ s^{-1}$, which lies within the range values discussed by Nill et al. (2018) for TrCel7A (reported range of values: 0.125-0.067 s^{-1} for desorption and 0.0007-0.14 $^{s-1}$ for decomplexation), in which several measured rate constants from multiple studies were reviewed and compared.

Comparing values for the binding rate constant is less straightforward, as its value depends on the definition of adsorption sites. The binding rate constant (which, in our case, is an average with no distinction between adsorption and complexation) is expressed in terms of moles of enzymes per volume of substrate per minute, which is the same basis used to describe the concentration of adsorption sites on the cellulose in the model. This definition makes direct comparisons with literature values challenging.

However, by assuming a particle with density including of 0.9 in solution and a loading between 2-10%, the desorption rate constant used in this study can be estimated at around 0.01-0.05 $\mu M^{-1}s^{-1}$, which is within the same order of magnitude reported for adsoprtion rates of 0.097-0.33 $\mu M^{-1}s^{-1}$ and complexation rates of 0.018-0.029 $\mu M^{-1}s^{-1}$ by Nill et al (2018). The slightly lower adsorption rates obtained can likely be explained by the fact that this parameter includes all enzymes, including some enzymes that bind and others that don't or may bind irreversibly to other parts of lignocellulosic biomass.

A7. Sensitivity Analysis

Sensitivity analyses were performed on relevant parameters for both cases with high cellulose accessibility (Dataset DS1, DAP mixed hardwood at 220°C for 7.8s) and low cellulose accessibility (dataset DS1, native mixed hardwood). Figure S7 shows the variation observed in 2h-glucose yield while changing the k_{des}, k_{ads} and D_{bulk} values by x10 or x0.1 times for each variable, while keeping other parameters equal to their initial values. Figure S8 represent the combined effect of change in fitted parameter τ and Mp on the predicted 2h-glucose yield for both substrates. For the substrate with low cellulose accessibility, the change in kinetic parameters have virtually no impact on the initial glucose rate, as the reaction rates are largely controled by internal diffusion limitations [Figure S6]. This effect is even more pronounced when the ratio of enzyme:initial binding sites is decreased, as the concentration gradient within the particle decreases further, slowing diffusion. In contrast, for particle presenting higher porosity, glucose release rates are strongly dependent to the desorption rate constant, while being relatively insensitive to the adsorption rate. In this case [Figure S6]. For both substrates, increasing the internal diffusion resistance by increasing the tortuosity lead to a shift in the fitted parameter M_p towards a larger value [Figure S7], which shows a proportional relationship between the optimal value of tortuosity and the value of M_p.

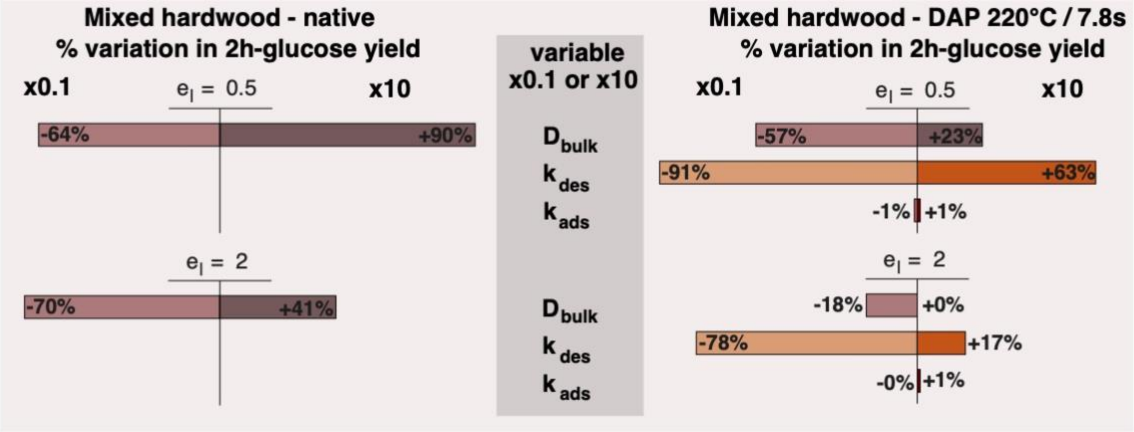

Figure S5. Sensitivity analysis for key model parameters. Both fitted parameters are kept constant at **τ = 2** and M_p = 755. Particle radius is assumed to be 25um.

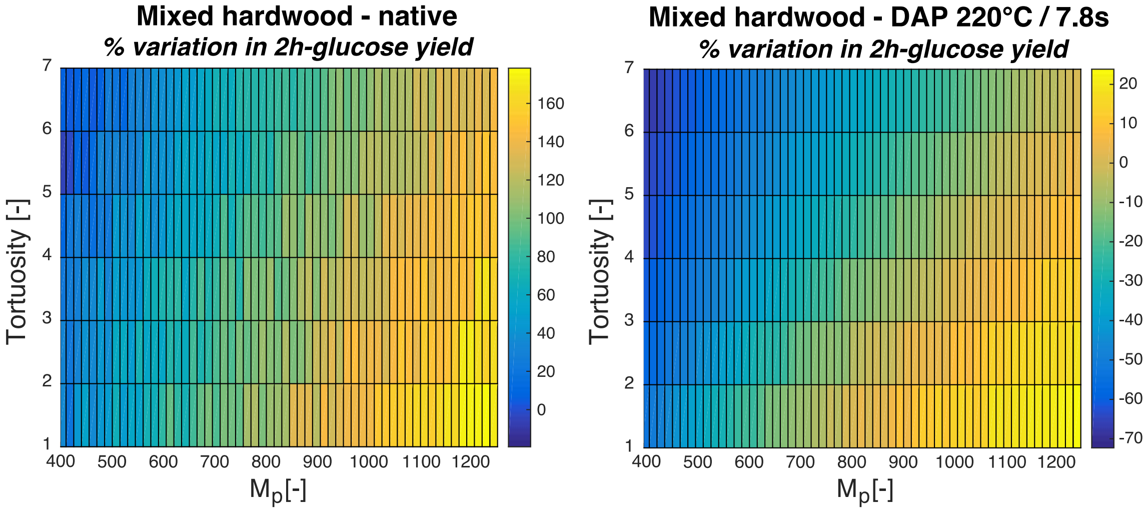

Figure S6. Sensitivity analysis for fitted parameter τ and M_p. The molar ratios of enzyme:initial binding sites were 6.7 and 0.6 for the native and DAP-pretreated substrate, respectively.

A8. Estimation of the number of accessible binding sites from porosity

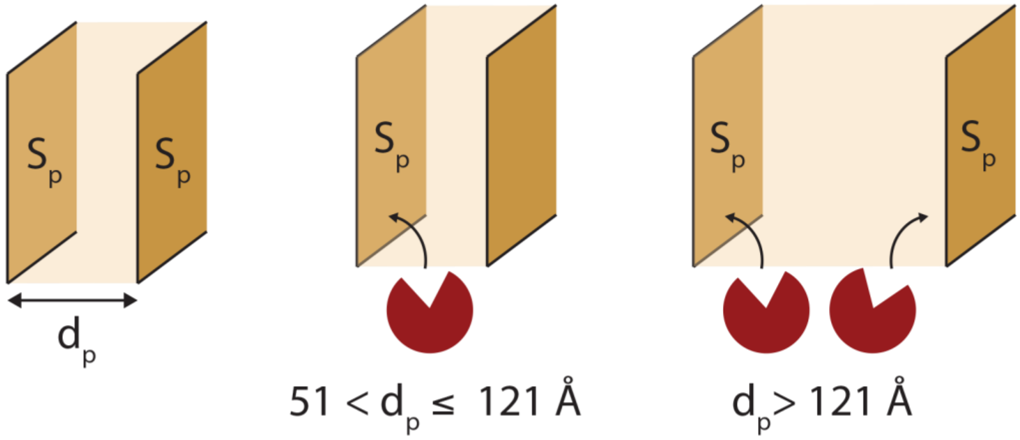

Figure S7. Schematic representation of the double-slit pore geometry, and how the pore's surfaces S_p are accounted for depending on the pore diameter d_p and the size of a cellulase.

The initial number of accessible binding sites is computed from the pore size distribution measured for a specific substrate using purely geometrical assumptions [Figure S1]. Based on the hypothesis that pores have a double-slit geometry (Stone and Scallan, 1968), the total pore surface S_{tot} can be computed from the pore volume V_p and the diameter/width d_p as follows,

$$S_{tot} = \frac{V_p}{d_p} \; for \; 51 < d_p \leq 121 \text{ Å} \quad or \quad S_{tot} = \frac{2V_p}{d_p} \; for \; d_p > 121 \text{ Å}.$$

Importantly, we assume that pores presenting a diameter smaller than that of a cellulase (i.e. smaller than 51A) are not accessible to cellulases and thus their pore surface is not accounted for in the total pore surface S_{tot}. In this study, a distinction is also made between pores that can accomodate 1 or 2 cellulases within their diameter by considering 1 or 2 pore surfaces within a slit geometry [Figure S1]. This surface is then converted into a number of cellulase adsorption sites by assuming (i) cellulose, hemicellulose and lignin are evenly distribute on the pore surface surface and (ii) that the footprint of a cellulase on the cellulose surface is equivalent to 5X5 nm (Bothwell, 1994).

A9. Boundary condition - derivation

Adopting Fick's law to describe the flux of enzyme $\boldsymbol{F}_E$ entering the particle from the bulk solution, the boundary condition at the outer radius of the cylindrical biomass particle is obtained by integrating this flux over the entire particle surface

$$\left.\frac{\partial C_E^F(r,t)}{\partial t}\right|_{r=R} = \left.\frac{\partial}{\partial t}\left[\frac{n_E^F(r,t)}{V_{Bulk}}\right]\right|_{r=R} = -\frac{1}{V_{Bulk}} \oiint_{S_{cyl}} \boldsymbol{F}_E \, dS$$

$$= -\frac{1}{V_{Bulk}} \int_0^{2\pi} \int_0^{L_A} D_E^{eff}(R,t) \nabla C_E^F(r,t) R d\varphi dz$$

$$= -\frac{2\pi R L_A}{V_{Bulk}} D_E^{eff}(R,t) \left.\frac{\partial C_E^F(r,t)}{\partial r}\right|_{r=R}$$

$$= -\frac{S_{cyl}}{V_{Bulk}} \varepsilon(R,t) D_E \left.\frac{\partial C_E^F(r,t)}{\partial r}\right|_{r=R}$$

Satisfying the latter condition ensures continuity at the solid-liquid interface.

A10. External vs. internal accessible cellulose surface

Assuming a cylindrical geometry for the biomass, the relationship between internal and external surface can be derived as follows. The external surface of a cylindrical particle $S_{cyl,ext}$ of radius R_{cyl}, not considering the edge surface, can be related to its volume V_{cyl}, and consequently its mass M_{cyl},

$$S_{cyl,ext} = \frac{2V_{cyl}}{R_{cyl}} = \frac{2M_{cyl}}{\rho_{cyl} R_{cyl}},$$

where ρ_{cyl} is the particle density including the void created by the pores. This lead to the ratio between external and internal surface being:

$$\frac{S_{cyl,int}}{S_{cyl,ext}} = \frac{S_{cyl,int} \rho_{cyl} R_{cyl}}{2M_{cyl}}.$$

As external surface is likely to be more important for particles exhibiting a low porosity and a small diameter, we consider here the extreme cases of a $10\ \mu m$ particle with a porosity of $6\ m^2/g$ of biomass (i.e. the typical accessibility of native mixed hardwood), leading to a

ratio of 29.5. In this extreme case, in which cellulose was also assumed to be evenly distributed in the particle, the error on the calculated accessible surface would be around 2%.

A11. Model implementation – verification and convergence

The convergence of the system against the discretization parameter n was first assessed to ensure reliable estimations of glucose release over time for relevant ranges of model parameters. As analytical solutions are not available for the system of equations considered here, the correctness of the implemented algorithm was evaluated by verifying proper conservation of enzymes over the course of the simulation.

The convergence of simulations can be evaluated by verifying that the conservation of the total number of enzymes in the system is conserved by calculating and verifying the following criterion η:

$$\eta(t) = \frac{|n_{E,tot}(t) - n_{E,0}|}{n_{E,0}} < 2\%$$

While increasing the discretization number improves accuracy, a trade-off value ensuring less than 2% deviation in the enzyme mole balance was chosen in order to alleviate computational cost and keep simulation time reasonable. For most systems considered here, this condition was already fulfilled with $n = 50$. Notable exceptions occurred for simulations that used low enzyme loadings ($e_l \leq 1$). In these cases, because the pore enzyme concentration tended to be particularly low due to significant surface adsorption, a finer grid was required to maintain numerical stability. Figure S1 shows convergence of the system as a function of the model parameters for a small particle, with different enzyme loadings. For lower enzyme loadings and/or higher biomass loadings (not pictured) the criterion was met only for a discretization number n above 2000. However, $n = 50$ was actually sufficient for most conditions. Notably, as a consequence of the finite time step

and the fact that results are essentially exact up to machine precision, η does not tend to zero, but to a finite value.

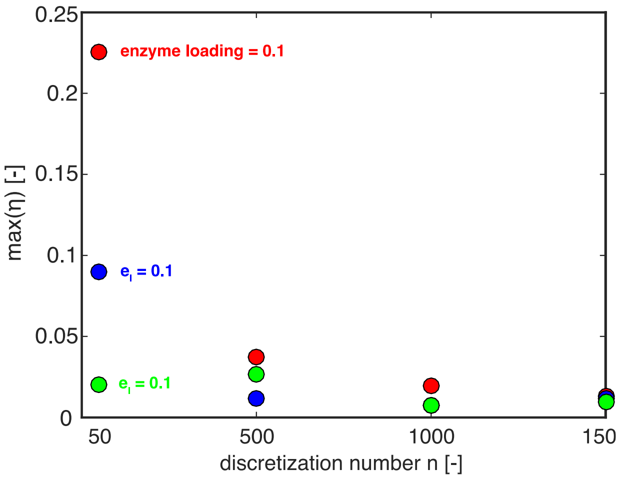

Figure S8. Convergence as a function of discretization number for particle exhibiting a radius of 10um and biomass loading of 1%.

A12. *Compositional Analysis*

Compositional analysis followed the LAP procedure published by NREL (NREL, Sluiter et al., 2012). Briefly, dried substrates were subjected to a two-step acid hydrolysis, starting with the incubation of samples (~0.3 g) in 7.5ml of acid solution (72% SA) for 2h at 30°C and 120 rpm, followed by dilution down to 3% sulfuric acid concentration before proceeding with the reaction at 121°C for 1h in an autoclave. Lignin was then separated from the soluble sugars by filtration and dried overnight at 105°C for quantification, while cellulose and hemicellulose contents were estimated through determination of sugar concentration in the filtrate by HPLC analysis (Bio-Rad Aminex HPX-87H column, 5mM H_2SO_4 mobile phase). The moisture content of all samples was initially determined by drying the wood particles overnight at 105°C and weighing the dried mass at room temperature after the sample was left for 2h in a desiccator.

A13. ***Pore size distribution***

Characteristics of the relevant probes employed in this study are summarized in Table S3.

TABLE S3

Molecular probe	Molecular weight[a] [g/mol]	Diameter[b] [A]
Glucose	180	8
P6000	6000	51
P35k	35000	121
P600k	600000	560

[a] From manufacturer (Merck)
[b] Extrapolated from Neuman et al., 2012, assuming an ellipsoidal shape for the polymer in solution

From the difference of concentration between the initial stock solution $C_{probe,init}$ and the solution left in contact with the wood sample $C_{probe,final}$, inaccessible pore volume for a given probe i was given by:

$$V_{inacc,i} = V_{probe} - V_{wood,wet} \cdot x_{wat} - \frac{C_{probe,init} \cdot V_{probe}}{C_{probe,final}}. \quad (1)$$

Measurement of the fiber saturation point (FSP) was performed using a probe that was assumed to be unable to penetrate into the pores. In this case, we used a probe with a size of 560 Å, and used the following formula to calculate the pore volume accessible to probe i:

$$V_{acc,i} = V_{inacc,560A} - V_{inacc,i}. \quad (2)$$

A14. ***Model - Error propagation***

To account for uncertainties associated with experimental measurements, and in particular those associated with the experimental determination of the pore volume distribution, a Monte-Carlo uncertainty analysis was performed in silico. To this end, the initial standard error associated with the accessible pore volume measured experimentally was sampled assuming a normal distribution using the function *normrnd* in Matlab. A

sampling number of 50 was found to ensure that the average pore volume calculated from the generated sampling distribution led to the correct average pore volume and associated standard error, while limiting computational cost. Simulations were then run for each of the generated data sets, which allowed for computing uncertainties on the predicted glucose yields.

References

Davis ME, Davis RJ. 2003. Fundamentals of Chemical Reaction Engineering. New York : McGraw-Hill

Kurašin, M.; Väljamäe, 2011. Processivity of Celliobiohydrolases is limited by the substrate. Journal of Biological Chemistry; 286(1):169–177.

Nill, J. E.; Karuna, N.; Jeoh, T. The Impact of Kinetic Parameters on Cellulose Hydrolysis Rates. Process Biochemistry 2018. https://doi.org/10.1016/j.procbio.2018.07.006.

Seiboth B, Ivanova C, Seidl-Seiboth V. 2011. Biofuel production – recent developments and prospects. Trichoderma reesei: a fungal enzyme producer for cellulosic biofuels. Croatia: InTech

Shi J, Ebrik MA, Wyman CE. Sugar yields from dilute sulfuric acid and sulfur dioxide pretreatments and subsequent enzymatic hydrolysis of switchgrass. Biores Technology. 2011;102(19):8930-8938

Sluiter A, Hames B, Ruiz R, Scarlata C, Sluiter J, Templeton D, Crocker D. 2012. Determination of structural carbohydrates and lignin in biomass. NREL/TP-510-42618, US National Renewable Energy Laboratory, Golden, Colorado.

Sun Q, Foston M, Meng X, Sawada D, Pingali SV, O’Neill HM, Li H, Wyman CE, Langan P, Ragauskas AJ, Kumar R. Effect of lignin content on changes occurring in poplar cellulose ultrastructure during dilute acid pretreatment. Biotech Bioeng. 2014;7(1):150-164
